# ZnO@Bi_5_O_7_I Heterojunction Derived from ZIF-8@BiOI for Enhanced Photocatalytic Activity under Visible Light

**DOI:** 10.3390/ma15020508

**Published:** 2022-01-10

**Authors:** Jijun Tang, Zhengzhou Duan, Qinyun Xu, Chuwen Li, Dongmei Hou, Guicheng Gao, Weiqi Luo, Yujia Wang, Yu Zhu

**Affiliations:** 1 School of Materials Science and Engineering, Jiangsu University of Science and Technology, Zhenjiang 212003, China; 200600002479@just.edu.cn (J.T.); duanzhengzhou@163.com (Z.D.); lichuwen1@163.com (C.L.); 18852894015@163.com (G.G.); lwq1791873658@163.com (W.L.); 2 Jiangsu Key Laboratory of Chiral Pharmaceuticals Biomanufacturing, College of Pharmacy and Chemistry & Chemical Engineering, Taizhou University, Taizhou 225300, China; xuqinyun2000@163.com (Q.X.); hdm3076391232@163.com (D.H.); ymjh_2020712840@163.com (Y.W.)

**Keywords:** ZnO, Bi_5_O_7_I, composite material, photocatalytic degradation, n–n heterojunction

## Abstract

In the study, ZIF-8@BIOI composites were synthesized by the hydrothermal method and then calcined to acquire the ZnO@Bi_5_O_7_I composite as a novel composite for the photocatalytic deterioration of the antibiotic tetracycline (TC). The prepared ZnO@Bi_5_O_7_I composites were physically and chemically characterized by X-ray diffraction (XRD), scanning electron microscopy (SEM), Brunauer–Emmet–Teller (BET) surface area, UV–Vis diffuse reflectance spectroscopy (DRS), emission fluorescence spectra, transient photocurrent response, electrochemical impedance spectra and Mott–Schottky. Among the composites formed an n–n heterojunction, which increased the separation efficiency of electrons and holes and the efficiency of charge transfer. After the photocatalytic degradation test of TC, it showed that ZnO@Bi_5_O_7_I (2:1) had the best photodegradation effect with an 86.2% removal rate, which provides a new approach to the treatment of antibiotics such as TC in wastewater.

## 1. Introduction

With the fast progress of industrial society, more and more antibiotics have been discharged into the nature together with sewage, leading to serious environmental pollution problems. As an infection-fighting and germicidal drug, tetracycline (TC) is resistant to degradation and easily induces microbial resistance in the environment, which is a major issue for environmental protection and sustainable development [1,2,3,4]. Semiconductor based photocatalysis technology as a green technology that does not use or generate hazardous substances that can remove persistent organic pollutants with low cost in an eco-friendly way has captured the attention of researchers around the world [5,6,7]. As one of the many well-known types of semiconductor materials, bismuth iodide oxide (Bi_x_O_y_I_z_) is a promising visible light responsive photocatalyst with narrow band gap, excellent photocatalytic performance, and good stability [8]. Bi_x_O_y_I_z_ has a particular alternating layer crystal structure with interleaved [Bi_2_O_2_]^2+^ layers, which provides an interior electrostatic field vertical to each layer and enhances the separation of photogenerated carriers [9]. The variations in the x, y, and z ratios lead to a significant difference in the photocatalytic property of Bi_x_O_y_I_z_. Within these Bi_x_O_y_I_z_ oxides, Bi_5_O_7_I possesses a modified valence band (VB) energy level with excellent photocatalytic activity to deliver more photogenerated holes, and Bi_5_O_7_I also shows the highest thermal stability [10,11,12,13,14,15]. Nevertheless, the photocatalytic properties of Bi_5_O_7_I still remain to be strengthened by increasing the band gap, carrier mobility, and improving the carrier-generated electron-hole pair separation efficiency [16]. In response to the shortcomings and defects of the Bi_5_O_7_I photocatalytic property, semiconductor materials such as Bi_5_O_7_I have been integrated to constitute heterojunction photocatalytic materials, which expand the absorption range of visible light of Bi_5_O_7_I by enhancing the separation efficiency of electron-hole pairs, and significantly increasing the photocatalytic performance and efficiency of Bi_5_O_7_I [17,18,19,20,21]. ZnO is a promising semiconductor material with band gap energy of 3.2 eV for photocatalytic applications [22,23,24,25]. Since ZnO exhibits a low refractive index, the light scattering effect of ZnO is comparatively small, which also facilitates the enhancement of photocatalytic efficiency. A metal organic framework (MOF) is a kind of crystalline porous material with high specific surface area consisting of inorganic metal centers and organic ligands [26,27,28,29,30,31,32]. It is often used to load various semiconductor materials to obtain more active photocatalytic materials. The photocatalytic materials obtained from MOF materials can acquire the desired composition and shape, and maintain the initial structural properties of porosity [33,34,35,36]. ZIF-8, as a kind of well-known MOF, not only has the features of a porous and adjustable structure, but also overcomes the shortcomings of MOF materials such as poor hydrothermal stability and structural collapse [37]. ZIF-8 has been used as a sacrificial template to obtain a ZnO semiconductor with a large specific surface area, high purity, high crystallinity, and regular morphology after removing C, H, and N elements from the skeleton structure by high temperature heat treatment [38,39].

In this article, a new type of n–n heterojunction photocatalysts was developed and synthesized using a straightforward hydrothermal process and calcination method. Related characterization and measurement techniques were applied to examine the crystal structure, chemical state, morphology, and photocatalytic properties of these binary composite materials. The ZnO@Bi_5_O_7_I composites demonstrated superior photocatalytic performance in the visible light-assisted decomposition of TC. The influences of various influencing factors were also studied including the initial tetracycline concentration, pH value, different cation concentrations, and photoinhibitors.

## 2. Experiment

### 2.1. Synthesis of BiOI

A total of 0.485 g Bi(NO_3_)_3_·5H_2_O was added into 40 mL methanol solution and dissolved completely, then 0.166 g of KI was solubilized in 10 mL deionized water before the KI solution was added into the methanol solution above with stirring. The mixture was then transferred to a hydrothermal reaction vessel (Xi’an YiBeiEr Instrument Equipment Co., Ltd, Xi’an, China) with a liner lined with Teflon and reacted at 180 °C for 2 h. When cooled to room temperature, the BiOI was acquired by centrifuging three times with deionized water and ethanol and dried at 80 °C under vacuum.

### 2.2. Synthesis of ZIF-8@BiOI

A series of ZIF-8@BiOI compounds consisted of different mass ratios of BiOI and ZIF-8. Different proportions of BiOI, 0.164 g of 2-methylimidazole, and 0.298 g of Zn(NO_3_)_2_·6H_2_O were dissolved in 40 mL methanol and then mixed at room temperature for 24 h. The mixture was centrifuged, washed with deionized water and ethanol and dried in an oven at 80 °C for 3 h to obtain ZIF-8@BiOI.

### 2.3. Synthesis of ZnO@Bi_5_O_7_I

Different ratios of ZIF-8@BiOI were put into a muffle furnace (Yixing Chuangzhuo furnace equipment Co., Ltd, Yixing, China), calcinated at 550 °C for 3 h and cooled to room temperature in a muffle furnace to obtain ZnO@Bi_5_O_7_I composites.

### 2.4. Characterization

The X-ray diffraction (XRD) data were collected on a Shimadzu XRD-6000 (Shimadzu instrument (Suzhou) Co., Ltd, Suzhou, China) apparatus. Scanning electron microscopy (SEM) was conducted on a Japan Electron JSM-6480 (Switzerland Wantong China Co., Ltd, Beijing, China) microscope to visualize the morphological appearance of the photocatalyst. Absorption spectra were registered on a Hitachi U4100 UV (Shimadzu instrument (Suzhou) Co., Ltd, Suzhou, China) detector. Fluorescence spectra were acquired on an FS5 (Edinburgh Instruments, EI, Edinburgh, UK) fluorescence spectrometer. Transient photocurrent measurements were conducted by an electrochemical workstation (Donghua DH-7000E) (Jiangsu Donghua Analytical Instrument Co., Ltd, Taizhou, China) equipped with three electrodes including an ITO electrode covering the specimens, and Pt and Ag/AgCl electrodes. For an individual working electrode, 5 mg of the specimen was distributed in 10 μL of nafion, and then 0.1 mL of anhydrous ethanol was added to generate a homogenized solution. Then, 40 μL of the above solution was dripped onto the ITO conducting glass. Aqueous 0.5 M Na_2_SO_4_ solution was employed as the electrolyte and exposed with a Xe lamp (Beijing puxie General Instrument Co., Ltd., Beijing, China) (250 W, λ > 420 nm). The impedance test was conducted in the range of frequencies from 0.1 Hz to 10 kHz, with amplitude an of 0.005 V, a quiet time of 2 s, and an initial potential of 0.071 V.

### 2.5. Photocatalytic Activity Test

TC was degraded by the photocatalyst when exposed to visible light. A 250 W xenon lamp (λ ≥ 420 nm) and UV cut-off filter were applied as the visible light source. The photocatalyst (50 mg) was distributed in 100 mL TC solution (10 mg/L) and the pH was measured to be approximately equal to 5 and then mixed in the dark for 30 min to establish the adsorption-desorption system. When the light was switched on, 4 mL of solution was retrieved every 5 min and isolated by centrifugation to acquire a clear solution. The TC concentrations in the solution were monitored by UV-Vis absorption at 357 nm.

## 3. Results and Discussion

### 3.1. Morphology and Structure

**Figure 1:**
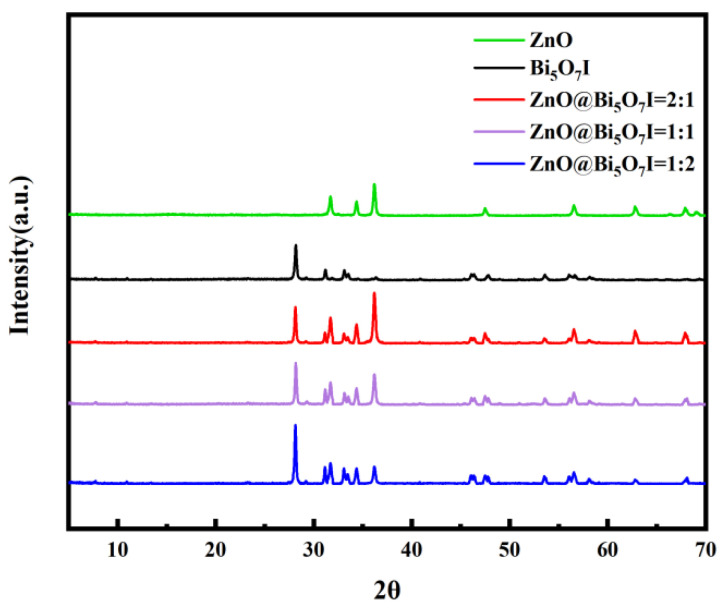
The X-ray diffraction spectra of the Bi_5_O_7_I, ZnO, and ZnO@Bi_5_O_7_I composites with different sample ratios.

The phase components and crystal structures of the synthesized ZnO, Bi_5_O_7_I, and ZnO@Bi_5_O_7_I materials were examined by X-ray diffraction analysis (Figure 1). The main peaks of ZnO were 2θ = 31.7°, 34.4°, and 36.2°, which were in accordance with the crystallographic planes of ZnO drawn by the standard JCPDS card 36-1451. In accordance with JCPDS card 40-0548, the predominant peaks of Bi_5_O_7_I were located at 2θ = 28.1°, 31.1°, 33.0°, 46.0°, and 53.5°, respectively. Typical diffraction peaks of Bi_5_O_7_I and ZnO can be noticed in ZnO@Bi_5_O_7_I, illustrating the two-phase composition in these composites. In comparison with pure Bi_5_O_7_I, the peaks of Bi_5_O_7_I in the composites were not shifted, indicating that the ZnO in the composites could not change the crystal structure of Bi_5_O_7_I.

**Figure 2:**
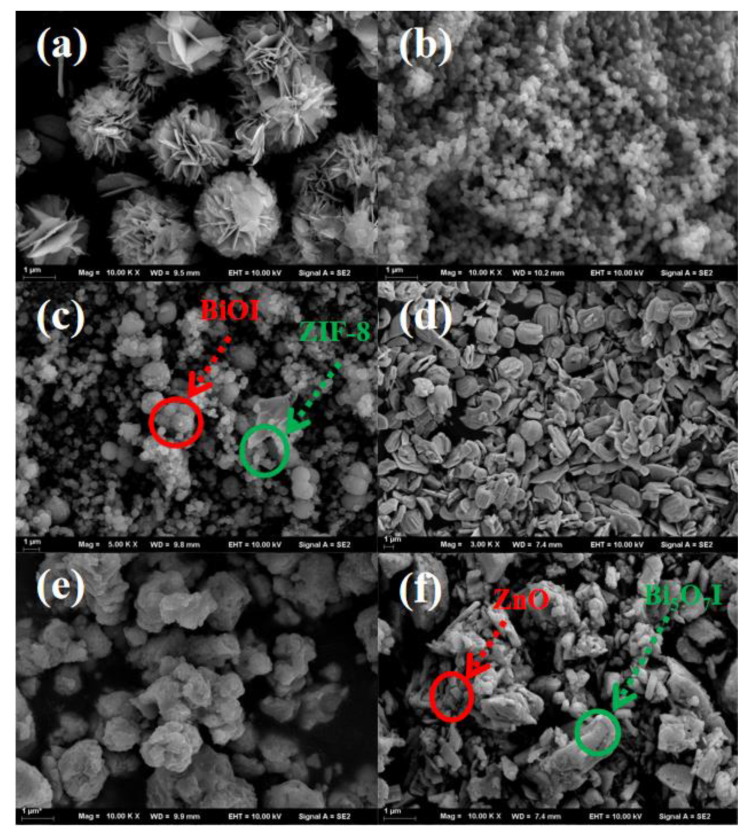
Scanning electron microscope image of (**a**) BiOI, (**b**) ZIF-8, (**c**) ZIF-8@BiOI (2:1), (**d**) Bi_5_O_7_I, (**e**) ZnO, and (**f**) ZnO@ Bi_5_O_7_I (2:1).

The morphologies of BiOI, ZIF-8, Bi_5_O_7_I, and ZnO were characterized by scanning electron microscopy (SEM). BiOI of a lamellar structure was stacked into a flower-like morphology (Figure 2a), while Figure 2b displays the diamond-shaped crystal structure of ZIF-8. The morphology of ZIF-8@BiOI was also collected to prove the successful growth of ZIF-8 on the surface of BiOI nanosheets (Figure 2c). Figure 2d demonstrates that Bi_5_O_7_I consisted of irregular, smooth-surfaced lamellar structures. It appeared from the SEM images of the composites (Figure 2f) that the distribution of ZnO on Bi_5_O_7_I was comparatively uniform. This facilitated the effective separation and transfer of photogenerated electrons and holes at the interface of ZnO and Bi_5_O_7_I and improved the photocatalytic degradation of ZnO@Bi_5_O_7_I.

**Figure 3:**
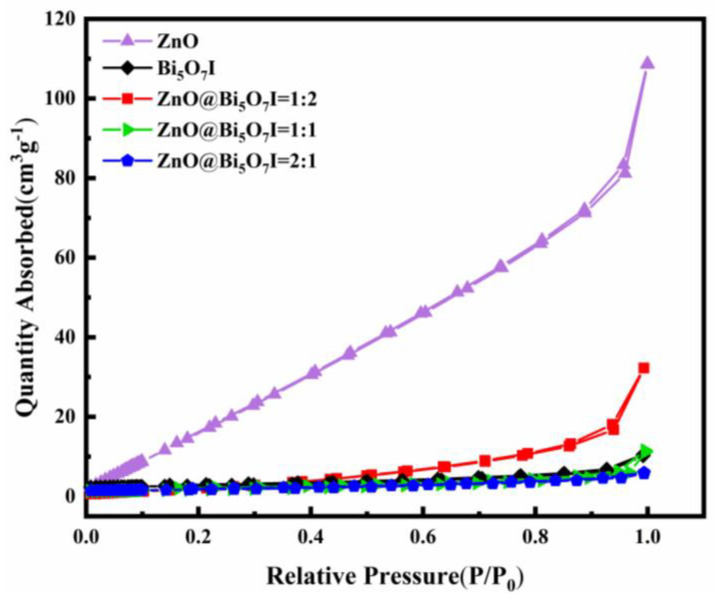
N_2_ adsorption and desorption isotherms.

Brunauer-Emmett-Teller analysis was used to characterize the specific surface area and porous properties of ZnO and Bi_5_O_7_I and the composite materials [40,41]. The specific surface area of ZnO was approximately 147.656 m^2^g^−1^, while the specific surface area of the ZnO@Bi_5_O_7_I composite was 5.6 (1:1), 22.8 (1:2), and 5.9 (2:1) m^2^g^−1^, respectively (Figure 3). In addition, the pore size of pure ZnO (2.2 nm) was smaller than that of ZnO@Bi_5_O_7_I = 1:1 (3.0 nm), ZnO@Bi_5_O_7_I = 1:2 (3.4 nm), and ZnO@Bi_5_O_7_I = 2:1 (2.5 nm) (Table 1). Nevertheless, the specific surface areas of the composites were larger than Bi_5_O_7_I (4.4 m^2^g^−1^). The recombination of semiconductors enhanced the specific surface area of the ultimate product, leading to the formation of multiple catalytic centers on the surface of ZnO@Bi_5_O_7_I, which increased the degradation intensity under visible light.

**Figure 4:**
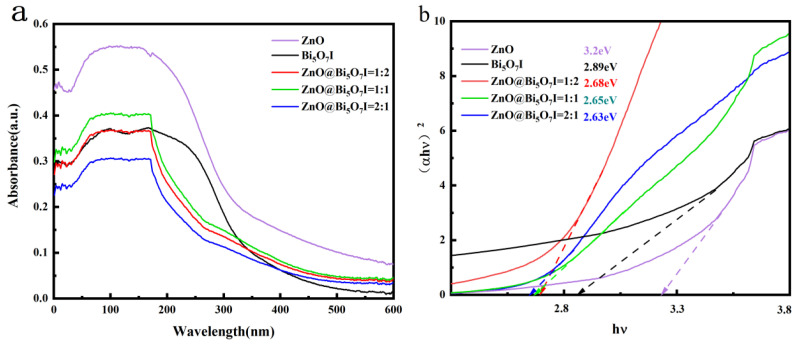
(**a**) UV-Vis diffuse reflectance spectrum and (**b**) band gap distribution of ZnO and Bi_5_O_7_I composite material magnification.

The prepared Bi_5_O_7_I, ZnO and composites are shown in the UV-Vis diffuse reflectance spectra, respectively (Figure 4). The absorption edges of Bi_5_O_7_I, ZnO, and ZnO@Bi_5_O_7_I were all at about 500 nm, indicating that both the monomer and composite were visible light responsive materials, as shown in Figure 4a. In accordance with the Kubelka-Munk curve and spectrum fitting, the band gap of Bi_5_O_7_I was 2.89 eV, while that of ZnO@Bi_5_O_7_I was 2.68 eV (1:2), 2.65 eV (1:1), 2.63 eV (2:1), respectively. As the band gap in the composites were reduced, electrons were more conveniently excited into photogenerated electrons in solution, which participated in the photocatalytic reaction and enhanced the photocatalytic degradation efficiency of the materials.

**Figure 5:**
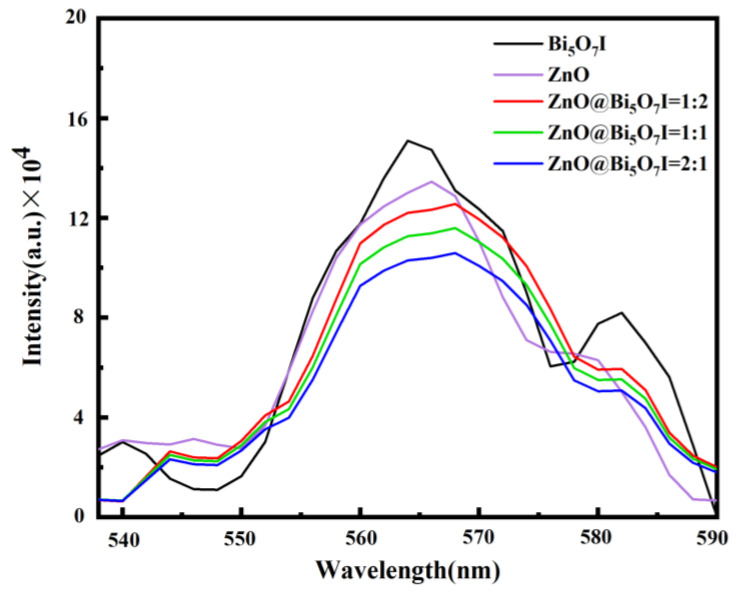
ZnO, Bi_5_O_7_I and emission fluorescence spectra of binary composites.

To further confirm the effective charge separation in the material, the samples were employed at the excitation wavelength of 340 nm by fluorescence spectroscopy. An emission peak was observed near 564 nm as a result of the recombination of the photogenerated electrons and valence band holes (Figure 5). The peak intensities of both ZnO@Bi_5_O_7_I (1:1) and ZnO@Bi_5_O_7_I (1:2) were higher than those of ZnO@Bi_5_O_7_I (2:1). The lower emission intensities of the composites were more conducive to charge separation, which suppressed the recombination of electron-hole pairs [40].

### 3.2. Photocatalytic Performance

The catalytic degradation abilities of pure ZnO, Bi_5_O_7_I, and ZnO@Bi_5_O_7_I samples were evaluated under visible light irradiation using TC as the contaminant. The following first-order kinetic equation was adopted to fit the experimental data: *ln* (*C*_0_/*C*) = *kt*, where *C*_0_ and *C* are the pollutant concentrations of the solution at time *0* and *t*, respectively, and *k* is the photocatalytic rate constant [41,42,43].

**Figure 6:**
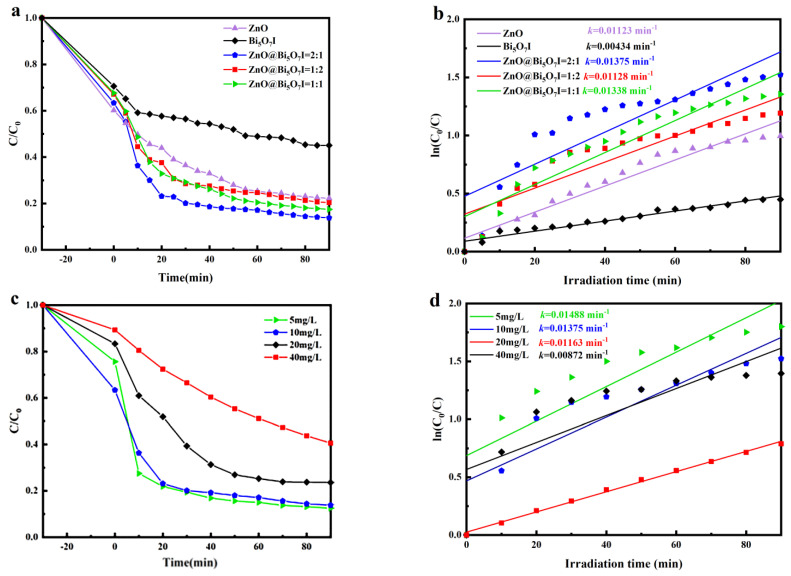
(**a**,**b**) ZnO@Bi_5_O_7_I samples at different ratios in TC 10 mg/L, and (**c**,**d**) photocatalytic degradation rates of TC in ZnO@Bi_5_O_7_I (2:1) with differing concentrations.

First, the photocatalytic performances of ZnO, Bi_5_O_7_I, and ZnO@Bi_5_O_7_I composites were assessed on TC degradation. In the dark, it was observed that the adsorption properties of thee ZnO, Bi_5_O_7_I, and ZnO@Bi_5_O_7_I composites were not significantly different. With the visible light, the removal rate of ZnO@Bi_5_O_7_I (2:1), ZnO@Bi_5_O_7_I (1:1), and ZnO@Bi_5_O_7_I (1:2) achieved 86.2%, 82.6%, and 79.6%, respectively (Figure 6a). After the first-order dynamics fitting, the *k* value of ZnO@Bi_5_O_7_I (2:1) was the largest (*k* = 0.01375 min^−1^), ZnO@Bi_5_O_7_I (1:1) = 0.01338 min^−1^, and ZnO@Bi_5_O_7_I (1:2) = 0.01128 min^−1^, respectively. It could be calculated that the *k* value of ZnO@Bi_5_O_7_I (2:1) was 3.16 and 1.22 times more than that obtained with Bi_5_O_7_I, ZnO, respectively (Figure 6b).

Figure 6c,d illustrates the effect of initial TC concentration on photocatalytic activity at initial concentrations of 5, 10, 20, and 40 mg/L. In the dark, the adsorption properties of photocatalysts in TC solutions with different concentrations were different: the absorption effect of 10 mg/L was the best, and that of 40 mg/L was the worst. In this photodegradation part, the degradation rate constants of ZnO@Bi_5_O_7_I (2:1) declined from 0.01488, 0.01375, 0.01074, and 0.01049 min^−1^ from 5 to 40 mg/L, respectively. When the TC concentration increased to more than 20 mg/L, the degradation rate constant decreased significantly. This can be accounted for by the fact that as the TC concentration increased, more TC molecules clustered around ZnO@Bi_5_O_7_I (2:1), prohibiting the exposure of visible light photons to the surface and thus reducing the possibility of producing active substances in the photocatalytic process [44].

**Figure 7:**
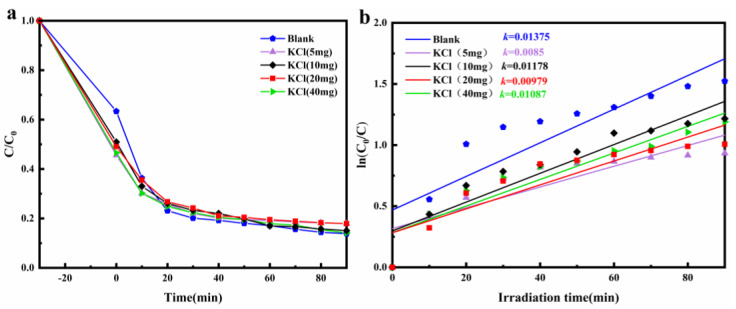
Photodegradation rates of TC (10 mg/L) in solutions containing ZnO@Bi_5_O_7_I (2:1) and different concentrations of KCl. (**a**) C/C_0_ vs. time, (**b**) ln(C/C_0_) vs. time.

As can be seen in Figure 7a,b, for the ZnO@Bi_5_O_7_I (2:1) composites, the addition of KCl increased the absorption of TC. Nevertheless, the effect on photocatalytic degradation was not significant, indicating that the composite was tolerant to the ions in the effluents.

**Figure 8:**
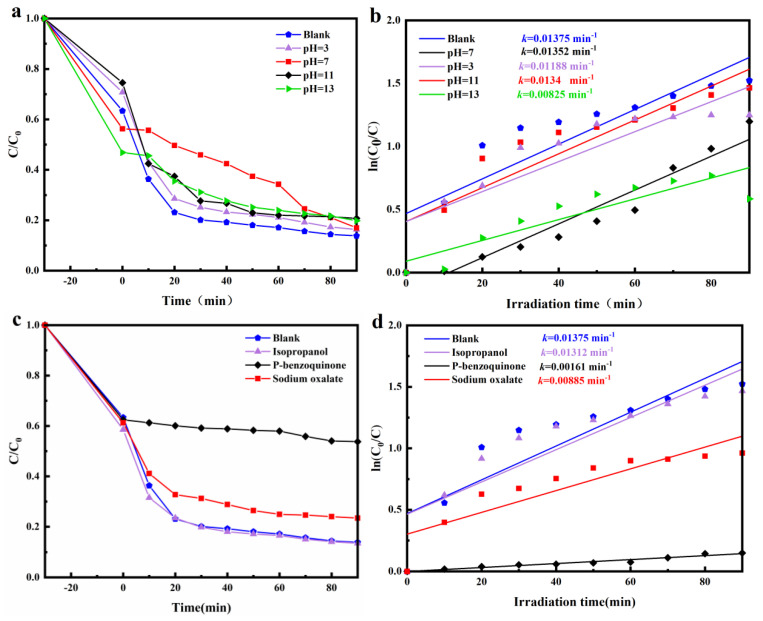
Photodegradation rates of TC (10 mg/L) in solutions containing ZnO@Bi_5_O_7_I (2:1). (**a**,**b**) Solutions with different pH, (**c**,**d**) Solutions with various organic compounds.

To examine the impacts of pH on the photocatalytic degradation of TC, we performed photocatalytic experiments in differing pH environments. The highest degradation rate was observed for the blank sample (pH = 5), and the degradation rates at different pH values were k_blank_ > k_pH=7_ > k_pH=11_ > k_pH=3_ > k_pH=13_ (Figure 8b), but TC removals at different pH did not differ significantly (Figure 8a). As reported, TC specie at strong alkali conditions were TCH^−^ and TC^2−^ [45]. The best adsorption effect was observed at pH = 13, which might improve the adsorption capacity of the composites through the electrostatic interaction of the photocatalyst surface with TC. Therefore, pH could affect the degradation efficiency of TC by changing the surface charge of the catalyst and its interaction [46,47].

To investigate the active species in the degradation reaction, trapping experiments of active species were carried out with the ZnO@Bi_5_O_7_I (2:1) composite. Isopropyl alcohol, p-benzoquinone, and sodium oxalate were commonly applied as OH, O_2_^−^, and photogenerated hole (h^+^) scavengers, respectively. Figure 8c,d indicates the role of these scavengers. The addition of p-benzoquinone and sodium oxalate produced remarkable effects on the photocatalytic degradation of TC, suggesting that both O_2_^−^ and photogenerated holes (h^+^) were active substances in the photocatalytic degradation mechanism. In contrast, the addition of isopropanol had insignificant effects on the photocatalytic degradation of TC, suggesting that OH was probably not the active substance in the mechanism of photocatalytic degradation.

**Figure 9:**
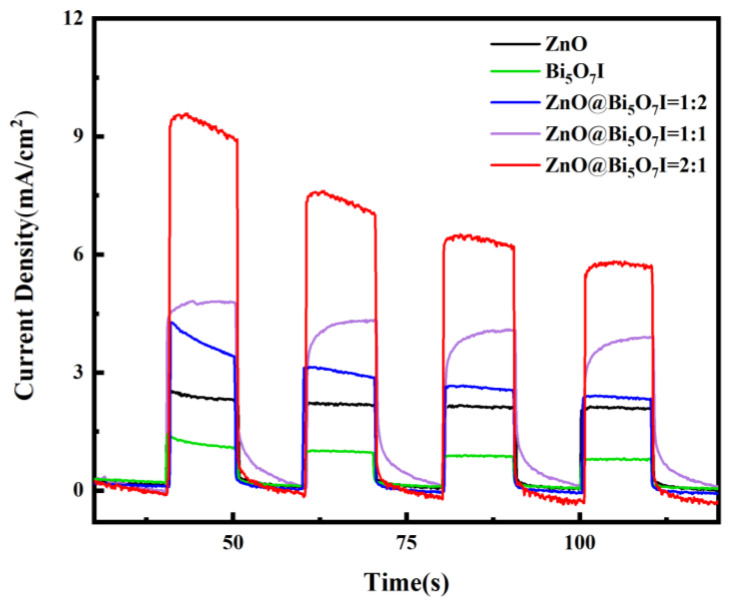
Photocurrent response plots of monomeric and binary complexes.

In order to examine the carrier transfer process and the separation process in photocatalysts, photoelectrochemical analyses were conducted. Figure 9 exhibits the transient photocurrent response curves of the original Bi_5_O_7_I, ZnO, and ZnO@Bi_5_O_7_I composites. Apparently, ZnO@Bi_5_O_7_I (2:1) displayed the most intense photocurrent response, which was much higher than that of the pristine Bi_5_O_7_I and ZnO, suggesting that the combination of ZnO with Bi_5_O_7_I significantly increased the charge separation and transfer.

**Figure 10:**
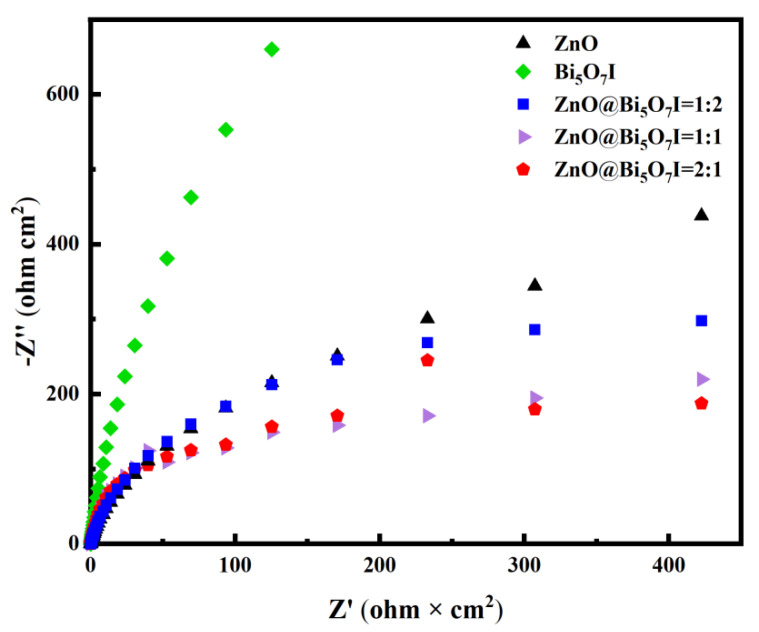
The Nyquist curves of the electrochemical impedance spectra displayed by the ZnO, Bi_5_O_7_I, and ZnO@Bi_5_O_7_I heterogeneous connections.

To further substantiate the mentioned results, the charge separation and transfer processes were investigated using the EIS Nyquist diagrams of monomers and composites. Figure 10 displays the Nyquist impedance plots of the monomer and composite. The arc in the Nyquist diagram mirrored the charge transfer kinetics, and the diameter of the semicircle denoted the charge transfer resistance [48]. ZnO@Bi_5_O_7_I (2:1) displayed the smallest arc radius, revealing that the transfer resistance of the ZnO@Bi_5_O_7_I (2:1) surface carriers was the lowest.

**Figure 11:**
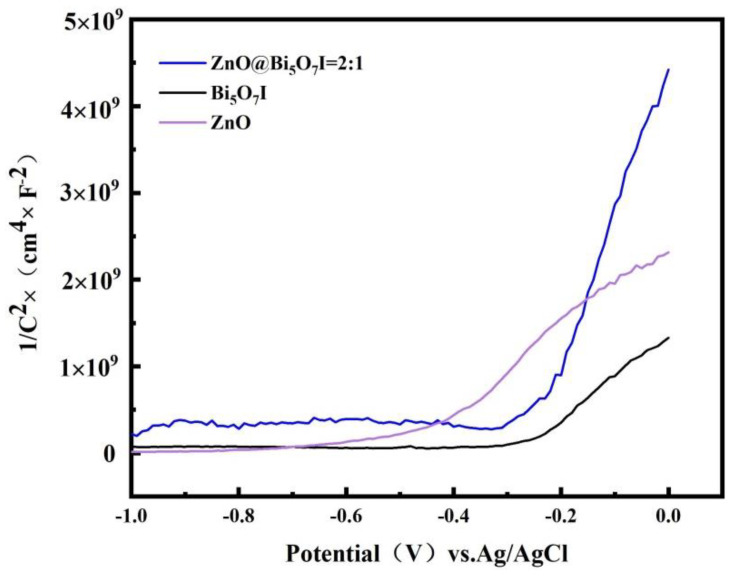
Mott-Schottky curve of Bi_5_O_7_I, ZnO, and ZnO@Bi_5_O_7_I.

Mott-Schottky (MS) plots of ZnO, Bi_5_O_7_I, and ZnO@Bi_5_O_7_I were evaluated, as shown in Figure 11. The positive slope of the Mott-Schottky diagram demonstrated that both ZnO and Bi_5_O_7_I were n-type semiconductors, which are a type of semiconductor in which the concentration of free electrons in the conductor was greater than the concentration of holes, and in which electron conduction was predominant. Based on the mentioned results and discussions, the formation of n-n heterojunctions at the interface of n-ZnO and n-Bi_5_O_7_I contributed to the effective separation and transfer of photogenerated carriers for TC photocatalytic degradation. The photogenerated electrons were switched from the E_CB_ (conduction band) of n-ZnO to the E_CB_ of n-Bi_5_O_7_I. Meanwhile, the photogenerated holes were diverted from the E_VB_(valence band) of n-Bi_5_O_7_I to the E_VB_ of n-ZnO. Upon exposure to simulated sunlight, the transferred electrons might be captured by molecular oxygen to form O_2_^−^ radicals, further oxidized and adsorbed on the photocatalyst surface [49,50]. Consequently, the photocatalytic efficacy of TC photocatalytic degradation was improved.

**Figure 12:**
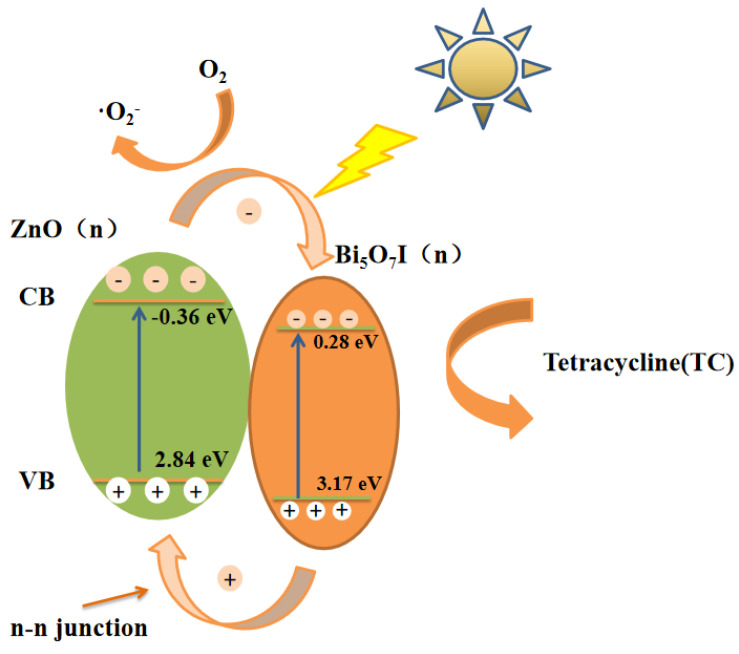
Energy band structure and charge separation diagrams of materials, and possible pathways for the photodegradation of pollutants.

Figure 12 shows a schematic diagram of the energy band structures of ZnO and Bi_5_O_7_I. Both ZnO and Bi_5_O_7_I were n-type semiconductors. The improved catalytic performance of ZnO@Bi_5_O_7_I heterojunction catalysts was attributed to the formation of heterojunctions between the two semiconductors and the interaction of the heterojunction interface. In accordance with the trapping experiments of active species shown in Figure 8c,d, the O_2_^−^ and photogenerated holes (h^+^) are known to be the main effect of the photodegradation of TC. It could be stated that the light irradiation forms electron-hole pairs for the prepared samples. Upon visible light irradiation, electrons leapt from the valence band of ZnO and gathered in its conduction band, and high-energy holes were generated in the valence band. Due to the high Fermi energy level of the conduction band in ZnO, the photogenerated electrons can easily be rapidly transferred from the conduction band of ZnO to the conduction band of Bi_5_O_7_I. The high-energy holes in the Bi_5_O_7_I valence band are transferred to the valence band of ZnO. This migration effect of photogenerated carriers can effectively suppress the compounding of photogenerated electrons and holes and improve the separation efficiency of the carriers.

The resulting cavities could generate O_2_^−^, and these abundant active species can oxidize organic pollutants under visible light irradiation pollutants to form inorganic small molecules under visible light irradiation. Thus, the mechanism of charge transfer of the proposed scheme well accounts for why h^+^ and O_2_^−^ were available to participate in the photocatalytic process. It can be observed that the combination of Bi_5_O_7_I and ZnO could dramatically increase the charge separation efficiency and enhance the photocatalytic degradation performance.

## 4. Conclusions

In this study, new types of ZnO@Bi_5_O_7_I materials were synthesized by calcination of ZIF-8@BiOI, and its properties were characterized. The ZnO@Bi_5_O_7_I (2:1) photocatalyst showed the best photocatalytic activity compared to pure ZnO, Bi_5_O_7_I and other ratios of ZnO@Bi_5_O_7_I composites. ZnO formed a heterojunction with Bi_5_O_7_I, which improved the electron-hole separation and prevented recombination, which can be explained by the photocatalytic mechanism. Consequently, the high catalytic performance of ZnO@Bi_5_O_7_I composites make them an excellent candidate for photocatalytic applications.

## Figures and Tables

**Table 1:** Specific surface, pore characteristics, and crystallite sizes of the samples.

Samples	SBET (m^2^g^−1^)	Pore Volume (cm^3^g^−1^)	Pore Size (nm)
ZnO	147.7	0.207	2.2
Bi_5_O_7_I	4.4	0.008	2.8
ZnO@Bi_5_O_7_I = 1:1	5.6	0.017	3.0
ZnO@Bi_5_O_7_I = 1:2	22.8	0.056	3.4
ZnO@Bi_5_O_7_I = 2:1	5.9	0.014	2.5

## Data Availability

Not applicable.
